# Influence of Iguratimod on Bone Metabolism in Patients with Rheumatoid Arthritis: A Meta-analysis

**DOI:** 10.1155/2022/5684293

**Published:** 2022-07-21

**Authors:** Li Deng, Fangling Yao, Feng Tian, Xiaowen Luo, Shenyi Yu, Zhenhua Wen

**Affiliations:** Department of Rheumatology and Immunology, Zhuzhou Hospital Affiliated to Xiangya Medical College, Central South University, Zhuzhou 412000, China

## Abstract

**Background:**

Influence of iguratimod on bone mineral density (BMD) and biomarkers of bone metabolism in patients with rheumatoid arthritis (RA) remains not determined. Accordingly, a meta-analysis was performed for systematical evaluation.

**Methods:**

Relevant randomized controlled trials (RCTs) were retrieved by searching of PubMed, Embase, Cochrane's Library, China National Knowledge Infrastructure (CNKI), and Wanfang databases. A random-effect model was used to pool the results.

**Results:**

In total, 24 RCTs including 2439 patients with RA contributed to the meta-analysis. Pooled results showed that compared to methotrexate alone, additional use of iguratimod 25 mg Bid for 12∼24 weeks significantly improved lumbar-spine BMD (mean difference [MD]: 0.12, 95% confidence interval [CI]: 0.04 to 0.20, *p*=0.002, *I*^2^ = 39%) in patients with RA. Moreover, treatment with iguratimod was associated with increased serum osteoprotegerin (MD: 180.36 pg/ml, 95% CI: 122.52 to 238.20, *p* < 0.001, *I*^2^ = 48%), and decreased serum receptor activator for nuclear factor kappa-B ligand (MD: −10.65 pmol/l, 95% CI: −15.59 to −5.72, *p* < 0.001, *I*^2^ = 53%). In addition, iguratimod was associated with increased bone formation markers such as the serum N-terminal middle molecular fragment of osteocalcin (MD: 4.23 ng/ml, 95% CI: 3.74 to 4.71, *p* < 0.001, *I*^2^ = 35%) and total procollagen type I amino-terminal propeptide (MD: 9.10 ng/ml, 95% CI: 7.39 to 10.80, *p* < 0.001, *I*^2^ = 86%), but decreased the bone resorption marker such as serum *β*-C terminal cross-linking telopeptide of type 1 collagen (MD: −0.18 pg/ml, 95% CI: −0.21 to −0.14, *p* < 0.001, *I*^2^ = 70%).

**Conclusions:**

Iguratimod could prevent the bone loss and improve the bone metabolism in patients with RA.

## 1. Introduction

Rheumatoid arthritis (RA) is a common chronic inflammatory disease characterized by polyarthritis [1, 2]. The primary symptom of RA at early stage is synovitis-related joint swelling [3]. With the progression of the disease, damage of articular bone and cartilage occurs, which further impairs the functional ability of the patients [4]. Despite of local bone erosion, which is considered as a central feature or RA and a key determinant of disease severity and poor functional outcome [5], patients with RA are also vulnerable to systemic bone loss, osteoporosis, and fractures [6, 7]. Indeed, a previous meta-analysis showed a pooled incidence of overall and fragility fractures were 33.0 and 15.3 per 1000 person-years, respectively [8]. Therefore, impairment of bone metabolism has been recognized as one of the important pathophysiological features of RA, which may adversely affect the functional ability and survival of these patients [9].

The mechanisms underlying the pathogenesis of bone loss in patients with RA are complicated [4]. Some studies have shown that proinflammatory responses presented as increased receptor activator for nuclear factor kappa-B ligand (RANKL) and decreased osteoprotegerin (OPG) in peripheral blood could contribute to the bone loss and osteoporosis patients with RA [10–12]. Moreover, patients with RA were also shown to have a reduced level of markers for bone formation, such as the N-terminal middle molecular fragment of osteocalcin (N-MID) and the total procollagen type I amino-terminal propeptide (T-P1NP), but an increased level of markers for bone absorption, such as the *β*-C terminal cross-linking telopeptide of type 1 collagen (*β*-CTX) [13], which collectively reflect the bone loss in RA. Iguratimod (IGU) is a novel small-molecule antirheumatic drug for patients with active RA [14]. Compared with conventional treatment, IGU combined with methotrexate (MTX) has been approved to have better efficacy and safety for RA patients [15]. Moreover, monotherapy with IGU has also been proposed as a potential alternative to MTX for the treatment of RA [16]. However, influence of IGU on bone metabolism in patients with RA remains largely unknown. Therefore, we performed a meta-analysis to systematically evaluate the influence of IGU on bone mineral density (BMD) and biomarkers of bone metabolism in patients with RA.

## 2. Materials and Methods

This systematic review and meta-analysis was performed in accordance to the PRISMA [17,18] (Preferred Reporting Items for Systematic Reviews and Meta-Analyses) statement and the Cochrane Handbook [19] guidelines.

### 2.1. Search Strategy

PubMed, Embase, and the Cochrane Library (Cochrane Center Register of Controlled Trials), China National Knowledge Infrastructure (CNKI), and Wanfang databases were systematically searched for relevant RCTs, using the combination of the following three groups of terms: (1) “Iguratimod” OR “alamode” OR “T-614”; (2) “bone” OR “osteoporosis” OR “rarefaction” OR “bone rarefaction” OR “bone mineral density” OR “BMD” OR “bone mass” OR “bone mineral content” OR “bone turnover” OR “bone resorption” OR “bone formation”; and (3) “random” OR “randomly”OR “randomized” OR “randomised.” The search was limited to studies in humans. We also analyzed reference lists of the original and review articles using a manual approach. Studies published from the inception of the databases until March 5, 2022, were retrieved.

### 2.2. Study Selection

Studies were included if they met the following criteria: (1) full-length articles published in peer-reviewed journals; (2) reported as RCTs with parallel design; (3) included adult patients with confirmed diagnosis of RA who were treated with background medications such MTX; (4) patients were randomly assigned to a treatment group of IGU, and a control group with placebo or no treatment; and (5) reported at least one of the following outcomes during follow-up, such as changes of BMD, OPG, RANKL, N-MID, T-P1NP, and *β*-CTX. Any parallel-group RCTs fulfilling the previously mentioned inclusion criteria were eligible for the meta-analysis, not limited to double-blind or placebo-controlled trials only. Reviews, observational studies, crossover studies, studies including patients without the diagnosis of RA, or studies did not report the outcomes of interest were excluded from the meta-analysis.

### 2.3. Data Extraction and Quality Assessment

Two authors independently performed the literature search, data extraction, and quality assessment according to inclusion criteria. If discrepancies occurred, they were resolved by discussion with the corresponding author. The following data was collected, such as the design characteristics, baseline characteristics of the included patients (age, gender, duration of RA, and background treatments), regimens of IGU and controls, follow-up duration, and outcomes reported. We used the seven-domain Cochrane Risk of Bias Tool [19] to evaluate the quality of the included studies, which include criteria concerning sequence generation, allocation concealment, blinding of participants and personnel, blinding of outcome assessors, incomplete outcome data, selective outcome reporting, and other potential threats to validity.

### 2.4. Statistical Analysis

Continuous variables were analyzed using mean difference (MD) and 95% confidence interval (CI). Changes of BMD, serum levels of OPG, RANKL, N-MID, T-P1NP, and *β*-CTX between baseline and endpoint in response to treatment of IGU as compared to controls were pooled separately in the meta-analyses. Cochrane's *Q* test was applied to evaluate the heterogeneity among the included studies. The *I*^2^ statistic was also determined, which indicates the percentage of total variation across studies that are due to the heterogeneity rather than chance [19, 20]. An *I*^2^ > 50% indicates significant heterogeneity among the trials. A random-effect model was used to pool the results since this model was considered to incorporate the potential between-study heterogeneity and could therefore minimize the influence of possible heterogeneity on the result [19]. Predefined subgroup analyses [19] were used to evaluate whether the difference of follow-up durations may affect the results. The median of the follow-up durations across the included studies was used as cutoff for defining subgroups. Potential publication bias was assessed with Egger's regression asymmetry test, or visual inspection of funnel plots if limited RCTs are included [21]. *p* values were two-tailed and statistical significance was set at 0.05. We used RevMan (Version 5.1; Cochrane, Oxford, UK) and Stata 12.0 software for the meta-analysis and statistical study.

## 3. Results

### 3.1. Search Results

**Figure 1:**
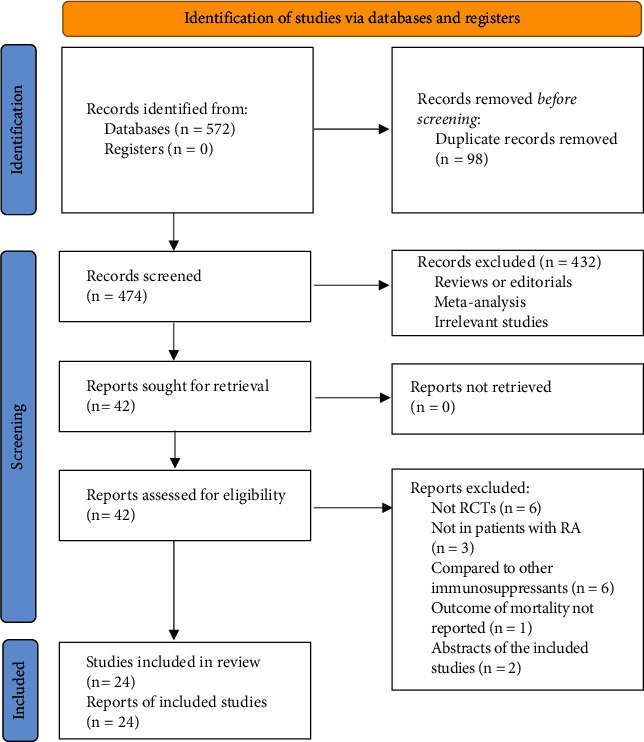
Flowchart of database search and literature identification.

A total of 572 articles were identified through database search, and 474 were retrieved after excluding the duplications. Subsequently, 432 were further excluded by screening of the titles and abstracts mainly because these studies were not relevant to the aim of the meta-analysis. Of the 42 potentially relevant articles for full-text review, eighteen studies were further excluded based on the reasons listed in Figure 1. Finally, the remaining 24 studies [22–45] met the inclusion criteria of the meta-analysis and were finally included for subsequent analyses.

### 3.2. Study Characteristics

Overall, 24 studies [22–45] including 2439 patients with RA contributed to the meta-analysis. The characteristics of the included studies are shown in Table 1. All of the included studies were open-label and parallel-group RCTs performed in China. All of the RCTs included patients with confirmed diagnosis of RA who were primarily treated with background therapy of MTX, hydroxychloroquine, or etanercept. Corticosteroids were not used in the patients of the included RCTs. For the three studies including patients with RA and osteoporosis, patients were additionally treated with oral calcium and vitamin D_3_ supplementation in two studies [31, 39], while no additional treatment for osteoporosis was applied for another study [38]. The sample sizes of the studies varied between 60 and 138. The mean ages of the patients varied between 45 and 73 years. The dosages of IGU were maintained as 25 mg bid in the intervention group, while no additional treatment was administered in the control group. The follow-up durations varied from 12 to 53 weeks.

### 3.3. Data Quality

The details of risks of biases of the included studies according to the Cochrane assessment tool are listed in Table 2. The details of random sequence generation were reported in five studies [22–25,32–34,37,38,40–42], and the details allocation concealment were not reported in any of the included studies. The details of withdrawals and dropouts were reported in all studies.

### 3.4. Influence of IGU on BMD in Patients with RA

**Figure 2:**
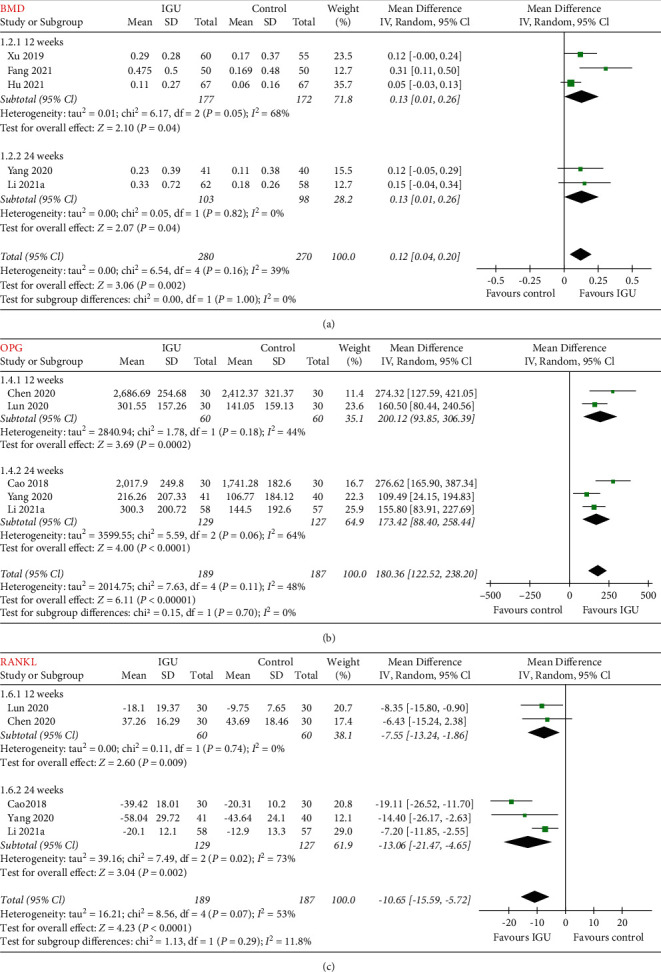
Forest plots for the meta-analyses evaluating the influences of IGU on BMD, serum OPG, and RANKL in patients with RA; (a) meta-analysis for BMD stratified by follow-up durations; (b) meta-analysis for serum OPG stratified by follow-up durations; (c) meta-analysis for serum RANKL stratified by follow-up durations.

Five of the included studies [31,36,38–40] reported the outcome of BMD, which were all measured as the lumbar-spine BMD. Pooled results of five studies [31, 36, 38–40] including 550 patients with RA showed that compared with control, treatment with IGU significantly improved lumbar-spine BMD (MD: 0.12, 95% CI: 0.04 to 0.20, *p*=0.002, *I*^2^ = 39%; Figure 2(a)). Subgroup analysis showed consistent results in studies with follow-up durations of 12 weeks (MD: 0.13, 95% CI: 0.01 to 0.26, *p*=0.04, *I*^2^ = 68%) and 24 weeks (MD: 0.13, 95% CI: 0.01 to 0.26, *p*=0.04, *I*^2^ = 0%; *p* for subgroup difference = 1.00; Figure 2(a)).

### 3.5. Influence of IGU on Biomarkers of Bone Metabolism in Patients with RA

**Figure 3:**
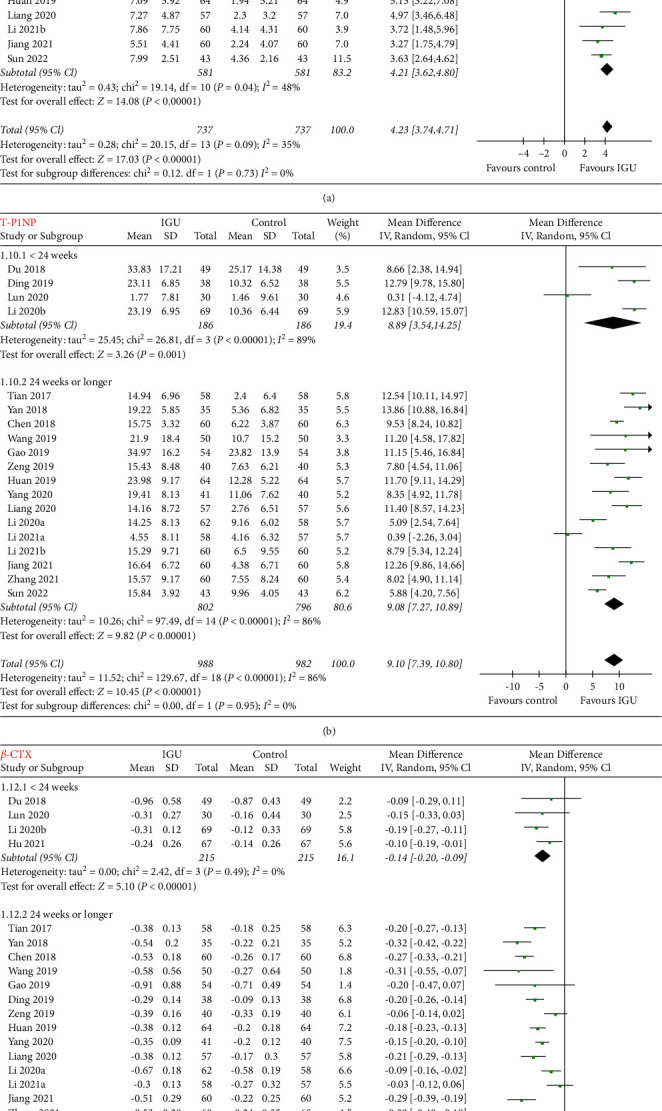
Forest plots for the meta-analyses evaluating the influences of IGU on serum N-MID, T-P1NP, and *β*-CTX in patients with RA; (a) meta-analysis for serum N-MID stratified by follow-up durations; (b) meta-analysis for serum T-P1NP stratified by follow-up durations; (c) meta-analysis for serum*β*-CTX stratified by follow-up durations.

Pooled results of five studies [23,33,37,38,42] including 376 patients with RA showed that IGU was associated with increased serum OPG (MD: 180.36 pg/ml, 95% CI: 122.52 to 238.20, *p* < 0.001, *I*^2^ = 48%; Figure 2(b)) and decreased serum RANKL (MD: −10.65 pmol/l, 95% CI: −15.59 to −5.72, *p* < 0.001, *I*^2^ = 53%; Figure 2(c)). Subgroup analysis showed consistent results in studies with follow-up durations of 12 weeks and 24 weeks (*p* for subgroup difference = 0.70 and 0.29, resp.). In addition, pooled results also showed that IGU was associated with increased serum N-MID (14 studies [22,24–30,32,34,35,41,43,45], MD: 4.23 ng/ml, 95% CI: 3.74 to 4.71, *p* < 0.001, *I*^2^ = 35%; Figure 3(a)) and T-P1NP (19 studies [22,24–30,32,34–38,41–45], MD: 9.10 ng/ml, 95% CI: 7.39 to 10.80, *p* < 0.001, *I*^2^ = 86%; Figure 3(b)), but decreased serum *β*-CTX (19 studies [22,24–30,32,34–38,40–42,44,45], MD: −0.18 pg/ml, 95% CI: −0.21 to −0.14, *p* < 0.001, *I*^2^ = 70%; Figure 3(c)). Subgroup analyses showed consistent results in studies followed <24 weeks, or in those with 24 weeks or longer (*p* for subgroup difference = 0.73, 0.95, and 0.21, resp.).

### 3.6. Publication Bias

**Figure 4:**
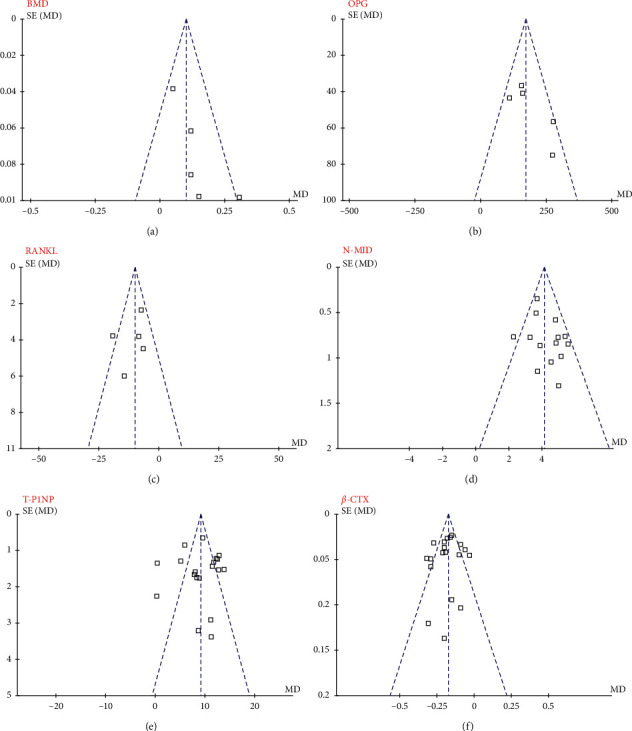
Funnel plot for the evaluation of publication biases of the meta-analyses; (a) changes of BMD; (b) changes of serum OPG; (c) changes of serum RANKL; (d) changes of serum N-MID; (e) changes of serum T-P1NP; (f) changes of serum *β*-CTX.

Forest plots for the meta-analyses for the influences of IGU on BMD, OPG, RANKL, N-MID, T-P1NP, and *β*-CTX were shown in Figures 4(a)–4(f). The plots were symmetrical on visual inspection, suggesting low risks of publication biases. The results of Egger's regression tests also suggested low risks of publication biases (*p* for Egger's regression tests all >0.05).

## 4. Discussion

In this study, by pooling the results of 24 RCTs, we found that, for patients with RA, additional treatment with IGU could significantly improve the lumbar-spine BMD as compared to controls with no additional treatment. Moreover, treatment with IGU in patients with RA was associated with significantly increased OPG and decreased RANKL in the peripheral blood. Additionally, IGU could also significantly increase serum markers of bone formation (N-MID and T-P1NP) and decrease the serum marker of bone resorption (*β*-CTX). Taken together, the results of the meta-analysis suggested that IGU could prevent the bone loss and improve the bone metabolism in patients with RA.

To the best of our knowledge, this may be the first meta-analysis which evaluated the influence of IGU on BMD and biomarkers of bone metabolism in patients with RA. Accumulating evidence suggests that besides synovitis-related joint damage and erosion of local bone, systemic bone loss and osteoporosis are also common in patients with RA [46]. More importantly, comorbidities related to the impairment of bone metabolism may increase the risk of fracture in these patients, which may further adversely affect the functional capacity and prognosis of patients with RA [47]. Therefore, effective prophylactic strategies are needed to prevent the bone loss and improve the bone metabolism in patients with RA [48]. The possible mechanisms for the systemic bone loss in patients with RA are complicated, including treatments related adverse influences, such as the use of corticosteroids, and inflammatory mediated mechanisms [48]. In our meta-analysis, we found that treatment with IGU significantly improved BMD as compared to control in patients with RA. These findings show that besides the validated therapeutic efficacy of IGU for relieving the symptoms of active RA, IGU may also exert additional benefit on bone metabolism in these patients. These findings are consistent with the results of several observational studies, which also suggested additional benefits of IGU on bone metabolism. A pilot nonrandomized study including 93 patients with RA showed that RANKL levels and the RANKL/OPG ratio significantly decreased in both serum and interleukin 1 beta-induced RA fibroblast-like synoviocytes after treatment with IGU [49]. Besides, another observational study also showed that IGU could stimulate bone formation in patients with RA, probably via regulating the RANKL/RANK/OPG system [50].

Subsequent meta-analyses further showed that the mechanisms underlying the potential preventative role of IGU on bone loss in patients with RA may involve the stimulating OPG, inhibiting RANKL, enhancing bone formation, and attenuating bone resorption. Subgroup analyses according to the follow-up durations of the studies showed consistent results. These findings are consistent with the findings of some preclinical studies. In a study of rat model of ovariectomy-induced osteoporosis, IGU was shown to inhibit RANKL-induced osteoclastogenesis and bone resorption in primary bone marrow mononuclear cells [51]. Moreover, a subsequent study suggested that IGU not only suppressed osteoclastogenesis by interfering with RANKL but also inhibited the bone resorption of mature osteoclasts in cultured bone marrow monocytes of the mice [52]. A recent in vitro study also showed that IGU significantly suppressed the dexamethasone-induced increase in osteoclasts, differentiation, and bone resorption activity, which also involved the regulation of the OPG/RANKL pathway [53]. Collectively, these findings indicated that iguratimod could preserve bone loss and improve bone metabolism in patients with RA. Besides, some recent studies also have highlighted the potential benefits of IGU on bone metabolism in other clinical circumstances other than RA. For example, in vitro and in vivo studies have consistently showed that IGU could effectively protect against cancer-induced bone pain and bone destruction, probably via downregulating interleukin-6 production in a nuclear factor-*κ*B-dependent manner [54, 55]. Moreover, an early study has also suggested a directly inhibitory role of IGU on osteoclast formation and function, which may also be a candidate mechanism underlying the preventative efficacy of IGU against bone destruction [56]. However, studies investigating the molecular mechanisms underlying the potential bone protective role of IGU remain rare, and future studies are warranted.

Our study also has limitations. First, all the included studies were from China. The potential benefits of IGU on bone metabolism should be validated in patients of other ethnicities. Second, the follow-up durations were from 12 to 53 weeks. The long-term influence of IGU on BMD and bone metabolism in patients with RA should be investigated in the future. Moreover, all of the included studies were open-label studies. The findings should be validated in large-scale placebo-controlled RCTs. In addition, in two of the included studies, patients of RA and osteoporosis were also additionally treated with oral supplementation of calcium and vitamin D_3_. Although patients of the IGU and control group both received the previously mentioned treatments, the possible imbalance of calcium and vitamin D_3_ status of the body may affect the results of BMD and markers of bone metabolism [57, 58]. Finally, lumbar-spine BMD was reported in all of the included studies. Influences of IGU on BMD of other sites should be evaluated.

To sum up, the results of the meta-analysis suggest that treatment with IGU in patients with RA is associated with improved lumbar-spine BMD, increased serum OPG, decreased RANKL, increased serum markers of bone formation (N-MID and T-P1NP), and decreased serum marker of bone resorption (*β*-CTX). These findings indicate that IGU may attenuate systemic bone loss and improve bone metabolism in patients with RA.

## Figures and Tables

**Table 1:** Characteristics of the included RCTs, BMD, OPG, RANKL, N-MID, T-P1NP, and *β*-CTX.

Author, year	Country	Study design	Diagnosis	Number of patients	Male	Age	Duration of RA	Background treatment	Intervention	Control	Follow-up duration	Outcomes reported
					%	years	years				Weeks	
Tian, 2017	China	R, OL	RA	116	45.7	51.1	9.1	MTX	IGU 25 mg bid	No treatment	24	456
Yan, 2018	China	R, OL	RA	70	32.9	56	11.4	MTX	IGU 25 mg bid	No treatment	24	456
Du, 2018	China	R, OL	RA	98	47.9	48.1	4	MTX	IGU 25 mg bid	No treatment	16	456
Cao, 2018	China	R, OL	RA	60	65	68	NR	MTX	IGU 25 mg bid	No treatment	24	23
Chen, 2018	China	R, OL	RA	120	35	45.8	7.1	MTX	IGU 25 mg bid	No treatment	24	456
Wang, 2019	China	R, OL	RA	100	41	61.4	9.3	MTX	IGU 25 mg bid	No treatment	24	456
Gao, 2019	China	R, OL	RA	108	42.6	47.9	3.1	MTX	IGU 25 mg bid	No treatment	24	456
Ding, 2019	China	R, OL	RA	76	42.1	72.7	8.7	MTX	IGU 25 mg bid	No treatment	16	456
Zeng, 2019	China	R, OL	RA	80	61.3	46.6	7.6	MTX	IGU 25 mg bid	No treatment	24	456
Xu, 2019	China	R, OL	RA and osteoporosis	115	NR	NR	NR	MTX	IGU 25 mg bid	No treatment	12	1
Huan, 2019	China	R, OL	RA	128	32.8	45.6	2.3	MTX	IGU 25 mg bid	No treatment	24	456
Yang, 2020	China	R, OL	RA and osteoporosis	81	21.1	45.9	5.5	MTX and HCQ	IGU 25 mg bid	No treatment	24	12356
Chen, 2020	China	R, OL	RA	60	55	70.3	NR	MTX	IGU 25 mg Bid	No treatment	12	23
Liang, 2020	China	R, OL	RA	114	43.9	53.9	7.3	MTX	IGU 25 mg bid	No treatment	24	456
Li, 2020a	China	R, OL	RA	120	55	46.2	4.1	MTX	IGU 25 mg bid	No treatment	53	12356
Lun, 2020	China	R, OL	RA and osteopenia	60	11.7	57.5	5.7	MTX	IGU 25 mg bid	No treatment	12	2356
Li, 2020b	China	R, OL	RA	138	41.3	65.5	5	MTX	IGU 25 mg bid	No treatment	12	456
Li, 2021a	China	R, OL	RA	115	53.9	46.3	5.5	MTX	IGU 25 mg bid	No treatment	24	12356
Fang, 2021	China	R, OL	RA and osteoporosis	100	NR	61.8	NR	MTX	IGU 25 mg bid	No treatment	12	1
Li, 2021b	China	R, OL	RA	120	65	52.9	5.3	MTX	IGU 25 mg bid	No treatment	24	45
Jiang, 2021	China	R, OL	RA	120	51.7	45.5	5.8	MTX	IGU 25 mg bid	No treatment	24	456
Hu, 2021	China	R, OL	RA	134	71.6	54.9	8.6	MTX or etanercept	IGU 25 mg bid	No treatment	12	16
Zhang, 2021	China	R, OL	RA and osteopenia	120	40	45.7	0.5	MTX	IGU 25 mg bid	No treatment	24	56
Sun, 2022	China	R, OL	RA	86	58.1	49	5.9	MTX	IGU 25 mg bid	No treatment	24	456

BMD: bone mineral density; OPG: osteoprotegerin; RANKL: receptor activator for nuclear factor kappa-B ligand; N-MID: the N-terminal middle molecular fragment of osteocalcin; T-P1NP: total procollagen type I amino-terminal propeptide; *β*-CTX: *β*-C terminal cross-linking telopeptide of type 1 collagen; RCT: randomized controlled trials; RA: rheumatoid arthritis; *R*: randomized; OL: open label; NR: not reported; MTX: methotrexate; HCQ: hydroxychloroquine; IGU: iguratimod; bid: twice daily.

**Table 2:** Quality evaluation via the Cochrane's Risk of Bias Tool.

	Random sequence generation	Allocation concealment	Blinding in performance	Blinding in outcome detection	Incomplete outcome data	Reporting bias	Other bias	Total
Tian, 2017	Low risk	Unclear	High risk	High risk	Low risk	Low risk	Low risk	4

Yan, 2018	Unclear	Unclear	High risk	High risk	Low risk	Low risk	Low risk	3

Du, 2018	Low risk	Unclear	High risk	High risk	Low risk	Low risk	Low risk	4

Cao, 2018	Low risk	Unclear	High risk	High risk	Low risk	Low risk	Low risk	4

Chen, 2018	Low risk	Unclear	High risk	High risk	Low risk	Low risk	Low risk	4

Wang, 2019	Unclear	Unclear	High risk	High risk	Low risk	Low risk	Low risk	3

Gao, 2019	High risk	Unclear	High risk	High risk	Low risk	Low risk	Low risk	3

Ding, 2019	Unclear	Unclear	High risk	High risk	Low risk	Low risk	Low risk	3

Zeng, 2019	Low risk	Unclear	High risk	High risk	Low risk	Low risk	Low risk	4

Xu, 2019	Unclear	Unclear	High risk	High risk	Low risk	Low risk	Low risk	3

Huan, 2019	Unclear	Unclear	High risk	High risk	Low risk	Low risk	Low risk	3

Yang, 2020	Low risk	Unclear	High risk	High risk	Low risk	Low risk	Low risk	4

Chen, 2020	Low risk	Unclear	High risk	High risk	Low risk	Low risk	Low risk	4

Liang, 2020	Low risk	Unclear	High risk	High risk	Low risk	Low risk	Low risk	4

Li, 2020a	Unclear	Unclear	High risk	High risk	Low risk	Low risk	Low risk	3

Lun, 2020	Low risk	Unclear	High risk	High risk	Low risk	Low risk	Low risk	4

Li, 2020b	Unclear	Unclear	High risk	High risk	Low risk	Low risk	Low risk	3

Li, 2021a	Low risk	Unclear	High risk	High risk	Low risk	Low risk	Low risk	4

Fang, 2021	Unclear	Unclear	High risk	High risk	Low risk	Low risk	Low risk	3

Li, 2021b	Unclear	Unclear	High risk	High risk	Low risk	Low risk	Low risk	3

Jiang, 2021	Low risk	Unclear	High risk	High risk	Low risk	Low risk	Low risk	4

Hu, 2021	Low risk	Unclear	High risk	High risk	Low risk	Low risk	Low risk	4

Zhang, 2021	Unclear	Unclear	High risk	High risk	Low risk	Low risk	Low risk	3

Sun, 2022	Unclear	Unclear	High risk	High risk	Low risk	Low risk	Low risk	3

## Data Availability

The data used to support the findings of this study are available from the corresponding author upon request.
